# Comparing ultrafiltration and equilibrium dialysis to measure unbound plasma dolutegravir concentrations based on a design of experiment approach

**DOI:** 10.1038/s41598-020-69102-y

**Published:** 2020-07-23

**Authors:** David Metsu, Thomas Lanot, François Fraissinet, Didier Concordet, Véronique Gayrard, Manon Averseng, Alice Ressault, Guillaume Martin-Blondel, Thierry Levade, Frédéric Février, Etienne Chatelut, Pierre Delobel, Peggy Gandia

**Affiliations:** 1 Department of Pharmacokinetics and Toxicology, Toulouse University Hospital, Toulouse, France; 2 INSERM, CRCT, Toulouse University, UPS, Toulouse, France; 3 INTHERES, INRA, ENVT, Toulouse University, Toulouse, France; 4 Toxalim, Toulouse University, INRA, ENVT, Toulouse, France; 5 Department of Infectious Diseases, University Hospital of Toulouse, Toulouse, France; 6 Inserm U1043 - CNRS UMR 5282, Toulouse-Purpan Pathophysiology Center, 31173 Toulouse Cedex, France; 7 Department of Biochemistry, Toulouse University Hospital, Toulouse, France; 8 INSERM UMR1037, CRCT (Cancer Research Centre of Toulouse), Toulouse University, UPS, Toulouse, France; 9 Department of Laboratory Medicine, GCS Ingres-Quercy, Montauban Hospital, Montauban, France; 10 Institut Claudius-Regaud, IUCT-Oncopole, Toulouse, France; 11 Laboratoire de Pharmacocinétique Et Toxicologie (Pharmacokinetics and Toxicology Laboratory), Centre Hospitalo-Universitaire Purpan (Purpan University Medical Centre), 330 avenue de Grande-Bretagne, 31059 Toulouse, France

**Keywords:** HIV infections, Antimicrobial therapy

## Abstract

Dolutegravir therapeutic drug monitoring (TDM) could be improved by measuring the unbound dolutegravir plasma concentration (Cu), particularly in patients experiencing virological failure or toxicity despite achieving appropriate DTG total plasma concentrations. Equilibrium dialysis (ED) is the gold standard to measure Cu, but ED is time consuming, precluding its use in clinical practice. In contrast, ultrafiltration is applicable to TDM, but is sensitive to numerous analytical conditions. In order to evaluate measurements of Cu by ultrafiltration, ultrafiltration conditions were validated by comparison with ED. DTG concentrations were measured by LC–MS/MS. Three ultrafiltration factors (temperature, duration and relative centrifugal force [RCF]) were evaluated and compared to ED (25/37 °C), using a design of experiment strategy. Temperature was found to influence Cu results by ED (*p* = 0.036) and UF (*p* = 0.002) when results were analysed with ANOVA. Relative centrifugal force (2000 g) and time (20 min) interacted to influence Cu (*p* = 0.006), while individually they did not influence Cu (*p* = 0.88 and *p* = 0.42 for RCF and time). Ultrafiltration conditions which yielded the most comparable results to ED were 37 °C, 1000 g for 20 min. Ultrafiltration results greatly depended on analytical conditions, confirming the need to validate the method by comparison with ED in order to correctly interpret DTG Cu.

## Introduction

Dolutegravir (DTG) is an efficient and well-tolerated antiretroviral currently used in antiretroviral therapeutic strategies^1,2^. Clinical studies have confirmed that the efficacy of DTG depends on drug plasma exposure (concentrations)^3,4^ and several efficacy-related targets have been proposed^5,6^. However, virological failures have been reported despite total concentrations (Ct) deemed to be efficient and close to the values recorded in HIV patients without virological failure^5–7^. Furthermore, the toxic neuro-psychiatric events described in a general HIV population cannot be explained by differences in DTG Ct^8^. Thus, therapeutic drug monitoring (TDM) which is currently based on Ct, reveals some limitations, and an alternative pharmacokinetic marker of exposure may be of interest in order to improve DTG-based strategies.

Dolutegravir (MW 419 g mol^−1^) is highly bound to plasma proteins (> 99%), binding mainly to albumin and, to a lesser extent, to the alpha-1-acid glycoprotein^9^. The amount of unbound DTG which is able to diffuse into tissues and across the cell membrane is relatively low^10^. It can therefore be assumed that any variation in the low amount of the unbound DTG form, which may not be detected by measuring the total plasma concentration of the drug, could explain the variability in pharmacodynamic effects such as virological failures or neuro-psychic events. Consequently, inter-individual variability of exposure to unbound DTG exposure (concentration) may explain differences in efficacy and/or toxicity to a greater extent than the total DTG concentration.

Before evaluating the pharmacokinetic/pharmacodynamic (PK/PD) relationship, it is particularly important to validate the method used to determine unbound DTG exposure (concentrations). Indeed, the unbound DTG concentration is directly related to the target virological concentration which yields DTG efficacy in the HIV compartment^11^. Equilibrium dialysis (ED) and ultrafiltration (UF) are the two main methods available to measure the concentration of the unboundDTG form. Because (1) non-specific binding (NSB) is low and (2) the unbound form is evaluated at binding equilibrium (BE), ED is considered the gold standard for evaluating concentrations of the unbound form of a drug^12,13^. However, this technique is time-consuming to perform and is not suitable for clinical trials or routine hospital laboratories^12,13^. In contrast, UF is a rapid, convenient method, that can be used to process many samples^12,13^. To date, UF is increasingly used to measure unbound exposure. However, despite high NSB and known analytical interferences, many authors still apply previously published UF technical conditions without validating these against ED^14,15^.

The aims of the current study were (1) to compare the characteristics of ED and UF approaches to explore DTG unbound exposures and (2) to determine the analytical conditions which validate the UF method when compared to ED.

## Materials and methods

### LC–MS/MS technique

Unbound and total dolutegravir concentrations were measured by a liquid chromatography—tandem mass spectrometry (LC–MS/MS) method which has been previously described^16^. Briefly, this method involves adding 200 µL of a precipitating reagent containing a deuterated internal standard to 50 µL of plasma, dialysate or ultrafiltrate prior to the LC–MS/MS analysis (ABSciex API-4500). Calibrator concentrations ranged between 50–10,000 ng/mL and 0.5–100 ng/mL for total and unbound DTG, respectively. Additional details about this LC–MS/MS method and performance are described in a previous study^16^. Briefly, inter-day and intra-day assays for unbound and total DTG concentrations (coefficient of variation) ranged from 1.28% to 7.09% for precision and from − 2.00 to + 13.87% for accuracy^16^. Precision and accuracy results, for both unbound and total concentrations, are detailed in supplemental data 1.Inter-day assays were performed over five different days (n = 15 samples for each corresponding quality control concentration) and intra-day assays were performed on five repeat samples for each corresponding quality control concentration.

### Sample preparation

Total DTG quality-control (QC) samples were prepared by diluting known concentrations of the working solution in plasma to yield final theoretical concentrations of 150, 1,500 and 7,500 ng/mL. For the unbound DTG QC, known concentrations of working samples were diluted in Sorensen buffer to yield final theoretical concentrations of 1.5, 15, and 75 ng/mL. More information on sample preparation is available in an earlier published study^16^.

### Measuring unbound DTG concentrations in plasma

#### Equilibrium dialysis

Equilibrium dialysis was performed in 1 mL Teflon cells using the Dianorm apparatus (Diachema AG, Zurich, Switzerland) with equilibrium achieved through Visking Dialysis Tubing membrane (molecular mass cut-off: 12,000-14000 Da, Medicell Membranes Ltd, London, UK). A 1 mL plasma aliquot was dialysed against 1 mL of Sorensen phosphate buffer (pH 7.4), in a chamber set to a nominal and controlled temperature of 37 °C and rotated at 12 rpm for 4 h (the Sorensen buffer preparation protocol is described in supplemental data 2). The resulting plasma and buffer dialysates were then promptly recovered from the Teflon cells and analysed by LC–MS/MS. Fifty microliters of dialysate was diluted with 200 µL of mobile phase containing 1 ng/mL of DTG-d4. Fifty microliters were injected into the LC–MS/MS system. Following dialysis, the remaining plasma was extracted with 200 µL of methanol containing DTG-d4 and then diluted in water (a fifth dilution). Analytical validation of DTG unbound fraction (fu, calculated as the ratio of unbound substance on total concentration; fu = Cu/Ct) through ED is described in detail in a previous study and summarised in a supplemental figure (supplemental data 3)^16^.

#### Ultrafiltration

Unbound-DTG determined using UF was measured in parallel to ED (on the same day). Prior to plasma UF, filters were pre-washed at 20 °C with LC–MS grade water for 20 min at 2000 g, according to the manufacturer's recommendations. Five hundred microliters of either a DTG spiked sample or patient plasma was incubated in a Centrifree (Millipore, Billerica, MA), with a molecular weight cut-off of 30,000 Da. The optimal UF conditions (temperature, time and relative centrifugal force [RCF]) were selected based on results from the reference method (ED at 37 °C). After UF, DTG was extracted from the plasma ultrafiltrate as previously described for the ED dialysate in the corresponding ED section above (see supplemental figure from supplemental data 3).

#### Equilibrium dialysis *vs.* ultrafiltration

Plasma samples used to compare both methods were spiked with 1 mg/L of DTG, which approximates concentration usually observed^7^. Following plasma spiking, samples were stored at 25 or 37 °C prior to analysis, depending on the temperature of the planned sample analysis (ED or UF, at 25 or 37 °C). We used a complete design of experiment (DOE) approach to predict the experimental conditions which would yield the most comparable readings between ED and UF. This procedure is useful to identify the level of interference from independent factors, such as temperature or from the interaction of two or more factors, such as time of centrifugation versus temperature^17^. The influence of three such analytical factors on UF results was evaluated. Two levels were studied for each factor: temperature (25 and 37 °C), RCF (1,000 and 2000 g) and centrifugation time (10 and 20 min). Eight different operating conditions were used to study these three parameters simultaneously, using five repeats per condition (refer to Table 1). The five repeats have been performed during a same assay. The sequence of analysis of the 8 UF conditions was randomised. In order to investigate the source of interferences on unbound DTG values, volume of ultrafiltration (V_uf_) was determined, as described in ‘Ultrafiltration method evaluation headings’. Results from UF were compared to ED results at 37 °C.

**Table 1:** Design of experiment comparing 8 conditions of ultrafiltration and 2 conditions of equilibrium dialysis for n = 5 samples each.

Condition	T (°C)	Time (min)	RCF (g)	Mean unbound fraction (%)	Unbound fraction, CV (%) [95 CI]	ANOVA	Dunnett test (*p* value)
Ultrafiltration						*p* < 10^–4^	
1	25	10	1,000	0.80	14.85 [14.74; 14.96]		0.044
2	25	10	2000	0.93	5.30 [5.26; 5.34]		< 10^−4^
3	25	20	1,000	0.87	7.63 [7.57; 7.69]		0.0262
4	25	20	2000	0.63	29.42 [29.25; 29.59]		0.999
5	37	10	1,000	0.58	7.72 [7.68; 7.76]		0.97
6	f37	10	2000	0.52	7.14 [7.10; 7.18]		0.46
7	37	20	1,000	0.65	7.46 [7.42; 7.50]		0.997
8	37	20	2000	0.85	6.45 [6.45; 6.49]		0.007
Equilibrium dialysis							
9	25	–	–	1.56	4.96 [4.89; 5.03]		< 10^−4^
10	37	–	–	0.64	6.61 [6.56; 6.66]		Reference group

*CV* coefficient of variation, *ANOVA* analysis of variance, *min* minutes; *g* gravitational constant, *T* temperature, *CI95* confidence interval, *RCF* relative centrifugal force.

The choice of the optimal UF conditions adopted was primarily based on results of statistical analyses (no statistically significant difference between UF and ED at 37 °C) and low dispersion of the results under the same condition (based on the coefficient of variation and 95% confidence intervals).

The data was compared to results obtained with ED at 37 °C, which was considered as the reference value, in accordance to previously published suggestions^12^.

#### Comparison of dialysate and ultrafiltrate matrix

As the same extraction procedure was applied to dialysate and ultrafiltrate samples, both matrices were compared. For that purpose, blank ultrafiltrates and dialysates were spiked with 1.5 and 75 ng/mL of DTG, respectively and extracted. The final DTG concentrations measured were subsequently compared.

#### Ultrafiltration method evaluation

The precision and accuracy of UF was determined using two kinds of samples (spiked and patient plasma samples). Inter-day assays were performed on spiked and patient samples on four different days, both by UF and ED (as reference method). The ratio of UF/ED fu (R_UF/ED_) was calculated for each sample. The accuracy, defined as (mean R_UF/ED_ − 1) × 100, and the precision, defined as (SD/mean R_UF/ED_) × 100 (SD = R_UF/ED_ standard deviation), were calculated. To date, there is no official guideline to evaluate accuracy/precision of the ultrafiltration method. A consortium of pharmaceutical companies recommends a precision fu CV < 30%. Based on FDA bioanalytical guidelines (for analytical procedures), a value of 100 + /− 20% was used as a criterion for accuracy. The accuracy of UF results was evaluated in relation to ED, which is the reference method to evaluate fu. It should be noted that the assumption of concordance between fu ED 37 °C/in vivo has not been verified using current procedures.

To evaluate DTG NSB, a LC/MS grade water solution was spiked with DTG at 1 mg/L and then serially diluted to yield samples of: 0.1, 0.01 and 0.001 mg/L. The dolutegravir concentration was measured in five replicate samples of each of these individual dilutions. Assays were performed at 25 and 37 °C in parallel. Non-specific binding was calculated as follows: NSB(%) = (1 − ultrafiltrate concentration/initial concentration) × 100. The weight of the ultrafiltrate obtained was used to calculate V_uf_. The density of the ultrafiltrate was extrapolated by comparison to the weight of 100 µL V_uf_ which did not contain any DTG.

In order to achieve V_uf_ lower than 50% of the initial plasma volume^12^, a short-duration (five minutes) UF assay was evaluated, independently of the DOE assays. Five plasma samples were each spiked with 1 or 0.1 mg/L of DTG. Ultrafiltration was then performed on five repeats of each of these concentrations at the following two conditions: (1) “short-duration” UF (37 °C, 1000 g, 5 min) and (2) UF condition providing comparable results to ED 37 °C (37 °C, 1,000 g, 20 min). V_uf_ was predicted according to the same method previously described. Both total and unbound DTG concentrations were measured.

#### Influence of confounding parameters on fu results

In order to explore the effect of a first freeze/thaw cycle on DTG/protein binding, assays were conducted on five DTG TDM samples. We evaluated any changes in pH, before and after thawing. A pH meter from Siemens RapidPOINT® 500 controller was used to measure pH. Unbound DTG concentrations were measured using the following UF protocol (37 °C, 1,000 g during 20 min; comparable to ED results).

Temperature (25/37 °C) is a parameter that may disrupt drug binding to plasma proteins^12^, as it is the case for calcium (Ca) binding to albumin^18^. This is particularly significant as a binding interaction (chelation) between Ca and DTG has been previously described^19^. In order to explain variations of DTG fu in ED according to temperature, pH and ionised Ca were measured on paired plasma samples (n = 5) at 25 and 37 °C. Plasma measurements were performed on the Siemens RapidPOINT® 500 controller. Sample temperature was maintained at 37 °C with a device routinely used for the determination of serum cryoglobulins. Ionised Ca is defined as free Ca which is able to bind to DTG.

#### Equilibrium dialysis and ultrafiltration of HIV patient samples

Once the conditions for achieving similar results between UF and ED were identified for spiked plasma, they were applied to HIV patient samples (n = 16). Immediately after being received at the laboratory, blood samples were centrifuged at 2000 g, + 20 °C for 15 min then the plasma was aliquoted and stored at − 20 °C until required for analysis.

Moreover, since samples (for analysis in a routine hospital laboratory) may not always contain the volume required (500 µl) to perform UF, the unbound form was also determined on duplicate 250 and 500 μL HIV patient plasma samples (n = 5). Assays for both volumes were performed in parallel on the same day.

This was a non-interventional study which did not require any additional procedures to be performed. Dolutegravir TDM and data collection were part of routine patient care. For these reasons, no Institutional Review Board or Ethics Committee approval was required, in accordance with French legislation governing biomedical research^20,21^.

### Statistical analysis

Analysis of variance (ANOVA) and a normality test were performed. Where the ANOVA test was statistically significant, Dunnett’s test was subsequently performed in order to identify a potential interaction. Results were expressed as median InterQuartileRange[25%;75%] and 95% confidence intervals). Whenever possible, results were associated with a paired t test and expressed as the mean and standard deviation (m ± sd). The relationship between two quantitative variables was assessed using Pearson's correlation test. The level of significance was set at *p* < 0.05. Statistical analyses were performed with the R software (3.5.1)^22^.

## Results

### Equilibrium dialysis *vs.* ultrafiltration

Results of the full design of experiment (DOE) analysis, comparing ED and UF are presented in Table 1. Similar results were obtained with an UF of 20 min at 1,000 g and at 37 °C (condition 7). In addition to the statistical outcome, this condition has also been chosen to stress the similarity of the result with ED at 37 °C and the low variability between results.

Temperature variations yielded statistically different free fraction (fu) results for both ED (m = 1.56% IQR[1.48;1.57] and m = 0.64% IQR[0.61;0.65], for ED at 25 and 37 °C, respectively; p = 0.036) and UF (m = 0.88% IQR[0.77;0.91] and m = 0.61% IQR[0.55;0.75] as well as for UF at 25 and 37 °C, respectively; p = 0.002), regardless of duration and RCF.

A comparison of UF temperatures revealed statistically significant differences in Vuf (m = 274 ± 28.2 µL and m = 298 ± 38.8 µL for UF at 25 °C and 37 °C, respectively; *p* = 0.036).

Furthermore, regardless of temperature conditions, the interaction of RCF (2000 g) and duration (20 min) of UF resulted in statistically different fu, compared to other conditions (*p* = 0.006 and < 10^–4^, at 25 and 37 °C, respectively) (Table 1). Ultrafiltrate volumes were higher in UF conditions performed at 2000 g and for 20 min, compared to the V_uf_ of other conditions (Table 2). However, when RCF and duration of centrifugation were analysed independently, no significant differences were observed in either V_uf_ (*p* = 0.588 for centrifugation time 10 vs. 20 min and *p* = 0.882 for RCF 1,000 vs*.* 2000 g) and fu results (*p* = 0.42 for centrifugation time 10 vs*.* 20 min and *p* = 0.88 for RCF 1,000 vs*.* 2000 g). In this instance, combined variations of duration and RCF (2000 g for 20 min) influenced V_uf_ and therefore the unbound fraction result, whereas individually, those conditions had no imapct.

**Table 2:** Results of mean ultrafiltrate volume under different temperature conditions.

Condition	Temperature	Volume (µL) mean; CV (%)	ANOVA	Dunnett test (*p* value)
1 (1,000 g 10 min)	25	252; 6.3	*p* = 0.006	0.00916
2 (1,000 g 20 min)	25	264; 9.9		0.03541
3 (2,000 g 10 min)	25	265; 13.1		0.03593
4 (2,000 g 20 min)	25	316; 11.3		Reference group
5 (1,000 g 10 min)	37	257; 6.1	*p* < 10^−4^	< 0.001
6 (1,000 g 20 min)	37	289; 7.7		0.0210
7 (2,000 g 10 min)	37	296; 17.0		0.0421
8 (2,000 g 20 min)	37	351; 8.4		Reference group

*ANOVA* analysis of variance, *g* gravitational constant, *CV* coefficient of variation..

In condition 8, the V_uf_ was higher compared to the other conditions (at both 25 and 37 °C; *p* = 10^−4^; Dunnet’s test with condition 8 as reference, *p* < 0.05; for condition 4, only a trend was observed with a mean difference of m = 0,035 CI_95_ = 0.053).

### Comparison of dialysate and ultrafiltrate matrix

Analytical extraction of DTG from the dialysate or ultrafiltrate was comparable for both low DTG spiked (m = 1.70 ± 0.05 ng/mL and m = 1.78 ± 0.03 ng/mL for dialysate and ultrafiltrate; *p* = 0.095) and high DTG concentrations (m = 80.83 ± 1.36 ng/mL and m = 81.93 ± 2.84 ng/mL for dialysate and ultrafiltrate; *p* = 0.578).

### Ultrafiltration method evaluation

Accuracy and precision of UF method were 121.5% and 27.2%.

Results revealed NSB ranging from 0.1% to 8.2% over the range of concentrations tested. NSB were comparable regardless of DTG concentration and/or temperature (*p* = 0.317).

Five minutes UF led to lower V_uf_ than a UF duration of 20 min (m = 224.5 mL IQR[212.5; 239.4] and m = 385.3 mL IQR[368.7; 417.3] for 5 min and 20 min respectively; *p* < 10^−4^). While a decrease in V_uf_ and a shorter duration of UF was associated with higher fu (fu m = 2.32% IQR[2.19; 2.45] and m = 1.83% IQR[1.71; 1.90] for 5 min and 20 min respectively; *p* < 10^−4^). The relative V_uf_ and fu increase, from 5 to 20 min-conditions, was 43 and 21%, respectively. The variation of fu over time, with the same temperature and RCF condition, was not linear. Between 5 and 20 min, fu decreases and between 10 and 20 min, fu increases (results detailed Table 1; comparison of results from conditions 5 and 7, *p* = 0.036).

### Influence of confounding parameters on fu results

An increase in pH was observed after one freeze/thaw cycle (proportion of pH increase m = 6.28% IQR[3.71; 8.61]; pH level m = 7.45 IQR[7.35; 7.55] and m = 7.92 IQR[7.75; 8.15], before and after thawing, respectively ; for pH level, mean and standard deviation of differences m = 0.47 sd = 0.28; *p* = 0.006). Unbound DTG concentrations measured before freezing ranged from 6.62 to 42.00 µg/L and remained unchanged after thawing (mean of relative differences m = 0.023; paired t-test *p* = 0.586).

At 25 °C pH was significantly lower, compared to measurements made at 37 °C (=+ 0.7% IQR[+ 0.5; + 0.8] for median pH increase; m = 7.53 IQR[7.45; 7.54] and m = 7.57 IQR[7.49; 7.60] for median pH in plasma at 25 °C and 37 °C respectively; *p* = 0.001 paired samples t-test).

Associated to lower pH, ionised Ca was significantly increased at 25 °C, compared to measurements made at 37 °C (m = −7.4% IQR[− 8.5; − 6.5] for median ionised Ca decrease ; m = 1.07 mM IQR[1.06; 1.11] mM and m = 1.00 mM IQR[0.98; 1.04] for median ionised concentration in plasma at 25 °C and 37 °C respectively; *p* = 0.003 paired samples t-test).

### Equilibrium dialysis and ultrafiltration on HIV patient samples

The patient dosage regimen was only based on 50 mg once a day*.* The seventh condition (following UF conditions: 37 °C with settled centrifugation at 1,000 g for 20 min) was then applied to HIV patient samples. Free fraction results did not differ statistically between UF (37 °C, 20 min and 1,000 g) and ED (37 °C) (m = 0.44 ± 0.06% and m = 0.47 ± 0.22% for ED and UF, respectively; *p* = 0.685). Comparison of 250 and 500 µL plasma volumes for UF did not reveal any statistically significant differences between fu results (m = 0.52 ± 0.09% and m = 0.60 ± 0.13% for 250 and 500 µL, respectively; *p* = 0.895, t-paired test). No relationship (*p* = 0.661) was observed between fu and Ct (range of Ct: 0.8 to 6 mg/L).

## Discussion

In our study, the two most commonly performed techniques for studying protein-drug binding were used and compared to determine the free DTG concentration. Equilibrium dialysis was used as the gold standard^12,13^ to evaluate and set the temperature, RCF and duration of centrifugation for UF.

Out of the three test parameters, temperature is the parameter most likely to influence fu results determined by UF^12,23–31^. However, because temperature could also modify the BE^26^, UF and ED were both compared in our study at two different temperatures, namely 25 and 37 °C. Since it allows approximation of the *in-vivo* condition, 37 °C is therefore considered as the reference temperature^12^. Consequently, 37 °C was the temperature selected for UF. Even if condition 4 (25 °C) provided results similar to those obtained with ED at 37 °C, this condition was rejected because (1) the temperature was not within the physiological range (37 °C), (2) the V_uf_ was > 60% of initial plasma volume (i.e. it could create a disruption in BE during UF^12^) and (3) this condition was associated with greater analytical variability.

Surprisingly and contrary to previous observations^27,31,32^ and results from our study (ED assays), increasing the temperature (from 25 to 37 °C) resulted in lower fu. Temperature is a parameter which conditions pH level and may thereby interfere with substance/protein BE^12^. In our study, pH fluctuations were not found to affect DTG binding. The increase in ionised Ca at 25 °C, compared to 37 °C may have altered the DTG/protein BE and consequently fu. Indeed, DTG like tigecycline^33^ chelates divalent cations (i.e. Ca, Mg, etc.). It may be hypothesised, that the increase in Ca in vitro may increase the amount of Ca bound to DTG. Consequently, DTG/protein BE is disrupted and the concentration of DTG-bound to protein is reduced. As the ED membrane between plasma/buffer compartments is permeable to DTG-Ca, this complex is also found in the buffer compartment. A new BE is achieved and the same amount of DTG/Ca is recovered on both sides of the membrane. The decrease in bound DTG associated with the increase of DTG in the buffer compartment may explain that fu at 25 °C is higher compared to 37 °C. In addition to the indirect effect observed in ED, temperature also affected the UF process. This effect was particularly noticeable with conditions 4 and 8 (2000 g/20 min at 25 and 37 °C, respectively; higher fu at 37 °C compared to 25 °C). Indeed, unexpected effects of temperature on fu at those “extreme” conditions of UF were observed (fu decreased at 25 °C and increased at 37 °C) compared to other UF conditions and ED. As neither pH, ionised Ca, nor NSB, could explain these results, temperature may have a direct influence on the UF process. According to Poiseuille's law and as illustrated by Cinar et al.^34^ with blood plasma, fluid viscosity decreases with increasing temperature. At 25 °C, condition 4 ("extreme" condition 2000 g/20 min) led to a larger V_uf_ compared to other conditions at the same temperature (1, 2 and 3). One can assume, as observed with 5/20 min UF assays, that such an increase in V_uf_ resulted in diluting the DTG contained in the ultrafiltrate. The fu decrease observed with condition 4, compared to other conditions, could therefore be explained by dilution of the ultrafiltrate. Conversely, at 37 °C, plasma viscosity is lowered and could explain even higher V_uf_ at condition 8 (2000 g 20 min), compared to the other conditions (both 25 and 37 °C). This V_uf_ greatly exceeded 50% of the initial plasma volume. The effect of such extreme UF on the BE is currently not well understood^12^. A disruption in DTG/protein BE could occur at condition 8, leading to an increased amount of DTG in the ultrafiltrate. As a result, the DTG fu also increases, as observed in our assays.

Besides temperature, RCF/duration interaction, which were poorly evaluated^25,27–29,31^, or a short duration (5 min) resulted in an increase of both fu and V_uf_. V_uf_ has been suggested as an indirect flag of the effect of UF on DTG/protein BE^12^. However, only sparse information is available relating to fu variations for V_uf_ > 50% (comparing with the initial plasma volume) and only high V_uf_ (> 80%) should be associated with BE disruption. Moreover, fluctuations in duration did not consistently have the same effect on V_uf_ and fu. Thus, variations of V_uf_/fu observed with DTG are comparable to those reported by Di et al.^28^ with vancomycin. These authors attributed this result to a BE disruption. Such a hypothesis is however not consistent with our results. Indeed, we found that fu decreased between 5 and 20 min while it increased between 10 and 20 min (at 1,000 g and 37 °C in both cases). The non-linear variation of fu over UF duration would therefore imply at least two processes (i.e. at first, a passage of unbound DTG with a high amount of DTG into the primary ultrafiltrate, subsequently followed by the passage of plasma ultrafiltrate, diluting the final ultrafiltrate). However, such results have never been described before and this mechanistic approach, as described in previous articles, is poorly compatible with BE from a thermodynamic point of view (BE reached within microseconds)^35^. Thus, no UF marker (e.g. V_uf_) predicting the conditions leading to identical results as ED at 37 °C for whole substances, has been identified to date. Even if the concordance of fu from ED and in vivo cannot be verified, ED is considered to be the gold standard to determine fu. Thus, for an accurate interpretation of unbound DTG concentration results, an experimental comparison of UF vs ED appears to be essential. The predicted effects of temperature, RCF and UF duration on fu results are summarised in Figure S1.

Based on these results, UF conditions were set at 37 °C, for 10 min at 2000 g. Besides, as the effects of some conditions, such as high pH observed during prolonged sample storage, were not explored in this study, it is recommended to use fresh plasma to study DTG fu. To complete results from DTG spiked samples, unbound forms from HIV patient plasma samples were also measured by both ED at 37 °C and UF at pre-defined conditions. Patient results validated UF to confirmed determination of the unbound form of DTG by UF. Moreover, two volumes (250 and 500 µL) were evaluated and validated for routine use of low volume samples.

The extraction procedure with the spiked dialysate buffer or ultrafiltrate did not reveal any interference from the solution matrices. This validation was necessary since their composition was relatively different, in particular the dialysate. Sorensen buffer mainly comprises a phosphate buffer whereas UF is a plasma ultrafiltrate, which therefore has a lower phosphate content^36^. However, this validation method is not described in the few articles comparing the two techniques^30^ although phosphate buffer could be a major limiting factor for LC–MS techniques due to an ion-suppression phenomenon^37^. In addition, in our method validation process, the first freeze/thaw cycle led to an increase in plasma pH. However, such an increase did not impair DTG/protein binding, in contrast to previous observations^12,33^. Non-specific binding of our UF method could be considered low, so there is no need to integrate a correction of the fu result^38^. This result could be attributed to the weak lipophilicity profile of DTG^9,35^. This result was comparable to that observed with our dialysis technique^16^. Moreover, NSB were consistent, regardless temperature (25 °C or 37 °C) and/or DTG concentration. Conditions used during UF allowed accurate determination of fu, compared to ED results obtained when applying recommendations from pharmaceutical company consortium (precision CV < 30%)^39^.

Despite promising results from our DOE analysis, our study did have some limitations. Indeed, we did not explore the effect of higher total DTG concentrations on UF results. However, since (1) fu results did not vary with total concentrations in HIV patient samples and (2) albumin physiological levels (close to 650 µM)^12^ are more than 50 times higher than the DTG total concentration (9.58 µM for the maximal concentration generally observed, close to 4 mg/L)^7^, protein-binding saturation at the standard DTG concentration is unlikely. Unfortunately, this result is not as powerful as performing a DOE evaluation and this interpretation should be considered with caution. Another limitation of our study was the evaluation of only two levels for each of the factors examined (temperature, RCF and duration). This approach does not allow the identification of non-linear relationships between parameters (e.g. temperature) and response (fu result), because results from only two levels per factor in a DOE cannot be extrapolated outside of the study conditions.

To date, few data are available on DTG unbound forms in HIV patients. Using an ED technique, at the same temperature, Imaz et al*.*^11^, found median fu values similar to ours (0.46% *vs.* 0.44%). In contrast, the Letendre et al.^14^ study which does not describe any device or analytical procedure used, observed a higher fu compared to Imaz et al*.*^11^ and our results (fu = 0.70% at the sixteenth week). Higher fu values from the Letendre et al*.*^14^ study were mainly driven by higher unbound concentration values compared to our results and results from Imaz et al*.*^11^, while their Ct values were comparable to our results. The discrepancy between published results is similar to that observed in our study between ED and UF without set conditions. This difference raises the issue of an appropriate interpretation of the unbound form of DTG. Indeed, unbound concentrations were measured and used (1) to explain DTG tissue diffusion (e.g. CSF and genital organs) and/or (2) to evaluate drug efficacy by comparing an unbound concentration to an in vitro inhibitory concentration (typically IC_50_)^11,14^. As a consequence, if the unbound concentration is over-estimated, particularly if using an UF method, the truly effective concentration (unbound) in an anatomical compartment could be misinterpreted. This misreading could introduce a bias in the PK-PD analysis and therefore in the selection of dosing regimen for antiretroviral strategies.

While unbound concentrations have been deemed effective concentrations, PK/PD study is still confined to a relationship between total concentration and viral load, both in terms of kinetics of decrease^3^ or rebound of viral load during virological failure^5^. This lack of interest in the unbound drug form mainly stems from pre-analytical and analytical constraints. Indeed, the measurement of unbound concentration requires a more technically intensive bioanalytical approach than determining total concentrations^12^. Where studies require the unbound concentration to be measured, the most convenient method, namely UF^13^ is predominantly chosen for the analysis. From an organisational point of view, this method is easy and quick to use. But, as stressed in our study, UF may exhibit several analytical disadvantages in the form of ultrafiltration issues involving set UF conditions or NSB^12^. As has been recommended by several authors^12,13,40,41^, it is imperative to validate UF conditions *versus* ED at 37 °C for each individual drug investigated. Many studies have to date explored the unbound form of ARVs, but technical procedures used to measure unbound concentrations are not always described and only a few of these studies compared UF to ED^13^. The lack of validation of the UF procedure may potentially introduce a bias when interpreting results or when making comparisons with previously published data. Despite a call from Boffito et al*.*^42^, guidelines on the bioanalytical validation of unbound drug measurements, such as those established by the FDA or EMEA for total drug concentrations^43,44^, still remain to be established. Recommendations should therefore focus on deficiencies which preclude standardised evaluation of unbound forms. This could be the first step in furthering PK-PD studies based on the pharmacologically active form of a drug.

## Conclusion

Our study defines pre-analytical and analytical conditions which facilitate a more uniform measurement of free DTG concentrations between UF and ED. Temperature, duration and RCF were identified as interfering factors, thereby highlighting the merits of simultaneously validating UF and ED protocols for the investigation of individual drugs.

## Supplementary information

Supplementary information 1.

Supplementary information 2.

Supplementary information 3.

Supplementary Figure S1.
