# Supplementary material for: Comparing ultrafiltration and equilibrium dialysis to measure unbound plasma dolutegravir concentrations based on a design of experiment approach
Source: Sci Rep. 2020 Jul 23;10:12265. doi: 10.1038/s41598-020-69102-y (PMC7378073; doi:10.1038/s41598-020-69102-y)
Supplement: Supplementary file 1 — Supplementary information 1. [file 41598_2020_69102_MOESM1_ESM.pdf]

Scientific Reports

Comparing ultrafiltration and equilibrium dialysis to measure unbound plasma dolutegravir concentrations based on a design of experiment approach.

David Metsu [1,2]; Thomas Lanot [1]; François Fraissinet [1]; Didier Concordet [3]; Véronique Gayrard [4]; Manon Averseng [1]; Alice Ressault [1]; Guillaume Martin-Blondel[5,6] ; Thierry Levade[7,8] ; Frédéric Février[9] ; Etienne Chatelut [2,10]; Pierre Delobel [5,6]; Peggy Gandia [*1,3]

Supplemental data 1: Precision and accuracy data on unbound and total concentrations method validation.

For total DTG concentrations, precision, for low (150 ng/mL), medium (1500 ng/mL) and high (7500 ng/mL) quality control (expressed as coefficient of variation [CV%]), were 1.73, 1.52, 1.28, respectively, and 5.77, 6.93, 6.23% for intra and inter-day, respectively.

For total DTG concentrations, accuracy, for low (150 ng/mL), medium (1500 ng/mL) and high (7500 ng/mL) quality control (expressed as coefficient of variation [CV%]) were +0.40, +13.87, +12.88 and -2.00, +10.60, +8.23% for intra and inter-day, respectively.

For unbound DTG concentrations, precision, for low (1.5 ng/mL), medium (15ng/mL) and high (75 ng/mL) quality control (expressed as coefficient of variation [CV%]) were 3.05, 4.72, 3.86 and 7.09, 4.38, 6.37% for intra and inter-day, respectively.

For unbound DTG concentrations, accuracy, for low (1.5 ng/mL), medium (15ng/mL) and high (75 ng/mL) quality control (expressed as coefficient of variation [CV%]) were +6.00, +5.33, +10.51 and +10.20, +1.20, +10.60% for intra and inter-day, respectively.
