# Supplementary material for: Comparing ultrafiltration and equilibrium dialysis to measure unbound plasma dolutegravir concentrations based on a design of experiment approach
Source: Sci Rep. 2020 Jul 23;10:12265. doi: 10.1038/s41598-020-69102-y (PMC7378073; doi:10.1038/s41598-020-69102-y)
Supplement: Supplementary file 2 — Supplementary information 2. [file 41598_2020_69102_MOESM2_ESM.pdf]

Scientific Reports

Comparing ultrafiltration and equilibrium dialysis to measure unbound plasma dolutegravir concentrations based on a design of experiment approach.

David Metsu [1,2]; Thomas Lanot [1]; François Fraissinet [1]; Didier Concordet [3]; Véronique Gayrard [4]; Manon Averseng [1]; Alice Ressault [1]; Guillaume Martin-Blondel[5,6] ; Thierry Levade[7,8] ; Frédéric Février[9] ; Etienne Chatelut [2,10]; Pierre Delobel [5,6]; Peggy Gandia [*1,3]

Supplemental data 2: Sorensen buffer protocol:

Sörensen buffer was prepared with sodium phosphate dibasic purchased from Merck (Darmstadt, Germany), potassium phosphate monobasic purchased from VWR international (Fontenay sous Bois, France), sodium hydroxide 32 % from Merck KGaA (Darmstadt, Germany), ultrapure water (Merck Millipore, Fontenay-sous-Bois, France) and adjusted to pH 7.4. To prepare one litre of Sorensen buffer solution, 9.07g of KH_2PO_4 was transferred to a one-litre volumetric flask and distilled water added to the 1 L mark. The solution was stirred with a magnetic bar and then vacuum filtered through a 0.22 µm aqueous filter. For 100 mL of the Na_2HPO4 solution, 1.18g of Na_2HPO4 was made up to a volume of 100 mL with distilled water, stirred and filtered as described for the previous solution. Eight mL of the Na_2HPO_4 solution was then mixed with the KH_2PO_4 solution to obtain a final volume of 1 litre. This solution was then transferred to a beaker and stirred. A 33% solution of NaOH was used to adjust the buffer to pH 7.4. Sorensen buffer was then stored for a maximum of one week at +4 °C.
