# Supplementary material for: Comparing ultrafiltration and equilibrium dialysis to measure unbound plasma dolutegravir concentrations based on a design of experiment approach
Source: Sci Rep. 2020 Jul 23;10:12265. doi: 10.1038/s41598-020-69102-y (PMC7378073; doi:10.1038/s41598-020-69102-y)
Supplement: Supplementary file 4 — Supplementary Figure S1. [file 41598_2020_69102_MOESM4_ESM.pdf]

Scientific Reports

Comparing ultrafiltration and equilibrium dialysis to measure unbound plasma dolutegravir concentrations based on a design of experiment approach.

David Metsu [1,2]; Thomas Lanot [1]; François Fraissinet [1]; Didier Concordet [3]; Véronique Gayrard [4]; Manon Averseng [1]; Alice Ressault [1]; Guillaume Martin-Blondel[5,6] ; Thierry Levade[7,8] ; Frédéric Février[9] ; Etienne Chatelut [2,10]; Pierre Delobel [5,6]; Peggy Gandia [*1,3]

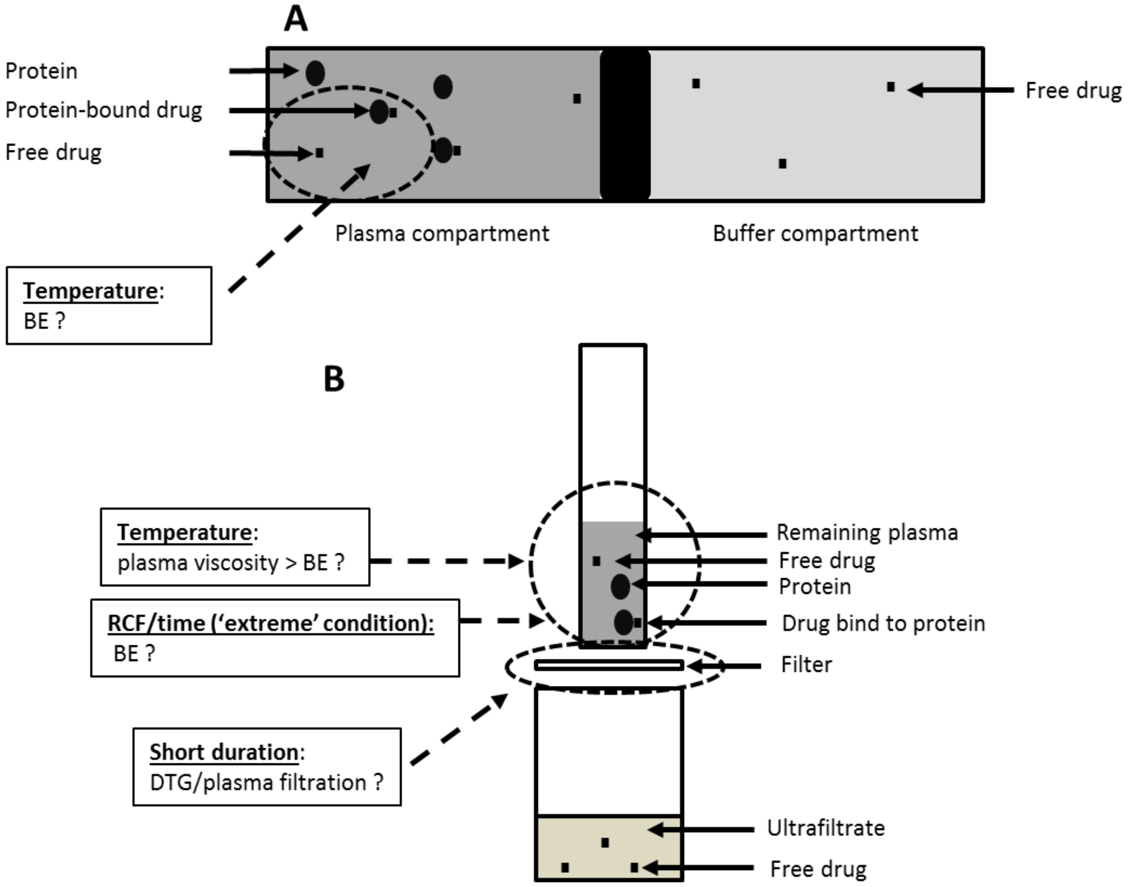

Supplemental figure S1: Assumed effect of temperature (A-Equilibrium dialysis and B-Ultrafiltration), relative centrifugal force and duration of ultrafiltration (B-Ultrafiltration) on fu results. Binding equilibrium depicted does not correspond to dolutegravir's binding equilibrium. BE: binding equilibrium; RCF: relative centrifugal force; DTG: dolutegravir
